# The wide-open doors to lexical access

**DOI:** 10.3389/fpsyg.2013.00471

**Published:** 2013-07-23

**Authors:** Jon A. Duñabeitia, Nicola Molinaro

**Affiliations:** Basque Center on Cognition, Brain and LanguageDonostia, Spain

Reading is an ability that appears simple and automatic to the experienced reader, in the same way that driving a car holds no mysteries for the practiced driver. However, most drivers would recall that the number of operations which needed to be learned to move the car smoothly seemed insurmountable during the first days of driving instruction. Nonetheless, as time passed by, thanks to repetition and practicing, and to the operations progressively becoming automatized, driving was no longer a challenge. Considering that in modern societies reading is typically acquired during early childhood, it is relatively implausible that we remember the hard moments we went through on the road to becoming fluent readers. Still, as is the case with driving, reading requires a substantial number of perceptual, attentional and mnemonic abilities, and a vast array of operations that can appear overwhelming to the neophyte until they become automatized.

Reading requires complex abstraction of the highly variable alphabetic visual input, which ultimately allows the access to the abstract orthographic categories that are in turn the door to the retrieval of phonological, morphological, lexical, and semantic representations. This stimulus-to-meaning mapping has to be robust enough to face font variability, handwriting styles, orthographic errors, contractions, and many other potential alterations in the input. This mapping poses the first paradoxical conundrum for the reader, who on the one hand has to be relatively “blind” to the obvious perceptual differences between multiple fonts, cases or handwriting of the same word (e.g., door, dOoR), and on the other hand needs to be “sighted” enough to detect basic perceptual differences between a given word and other similar items (e.g., door, deer, odor, dear).

The time window in which a given letter string passes from being a mere sequence of printed curves and strokes to acquiring the word status takes around one third of a second. In that fraction of a second the expert reader manages to access the meaning represented by the written symbolic and arbitrary graphic patterns. This phenomenon represents a model of human abstract symbolic thinking, since there is no direct relation between the meaning of a word and its written form. If we consider the concepts of a *door* and a *window*, it seems relatively straightforward to define the semantic relation between them. However, from a linguistic perspective there is no physical or functional relation between the two written codes *door* and *window*. How is it then possible that readers are able to compute the semantic relation between these two written codes through a simple eye fixation of 250 ms? What does reading imply for the human brain? And where and when in the brain does reading take place?

The answers to these questions are still controversial. Nonetheless, in recent years the neurocognitive literature has provided the grounds for constructing the perfect test scenario to help solve this issue. What, where and when? Neuroimaging and behavioral methods have demonstrated that reading implies a complex pattern of feed-forward and feedback interactive activations flowing along the visual recognition system, mainly in ventral regions of the left temporal lobe. Still, the precise way in which all the intermediate representations between a physically concrete printed stimulus and the mentally stored abstract lexico-semantic representation are activated is still debated and needs to be further explored.

The present Research Topic aimed to create a landmark forum in which experts in the field define the state of the art and future directions. A total of 10 excellent articles have been compiled (six Original Research articles, three Review articles and one General Commentary). Su et al. (2012) open the section of Original Research articles with an experiment using ERPs to test the interactions between graphemic similarity, position of the radicals of Chinese characters and lexical access. Next, Sliwinska et al. (2012) present the readership with a study using chronometric TMS devoted to better characterizing the role of the supramarginal gyrus in phonological processing, and ultimately, in visual-word identification. In the third article, Grossi et al. (2012) present an ERP study exploring the interactions between bilinguals' linguistic experience and orthographic and lexico-semantic effects associated with cross-language orthographic neighborhood effects in two groups of English-Welsh bilinguals. Hand et al. (2012) present an article exploring the early interactions between the orthographic constraint imposed by word-initial letters and context-based predictability effects using eye movement tracking techniques. A similar rationale is followed in the article by Lee et al. (2012), offering electrophysiological data regarding interactions between contextual information and early orthographic processing. Kinoshita and Norris (2012) provide the last Original Research article summarizing recent findings from the visual-word recognition domain and proposing an interpretation of masked priming based on the Bayesian Reader account that explains some controversial task-dependent effects. The Research Topic then continues with three Review articles and one General Commentary. Van Assche et al. (2012) offer an outline of recent data demonstrating that lexical access is language-non-selective in bilinguals, both at the level of recognizing words in isolation and at the level of recognizing words in sentence context. Hyönä (2012) presents an overview of the findings on compound word identification, and provides a physiologically valid for the way in which polymorphemic words are processed in alphabetic languages, based on visual acuity principles. Amenta and Crepaldi (2012) offer the last Review article, which is also related to the processing of polymorphemic words. They summarize benchmark morphological processing effects and set the scenario for future experimental and theoretical work by highlighting the most consistent and inconsistent findings. The General Commentary by Koester (2012) extends some of the issues raised by Amenta and Crepaldi (2012), and raises other concerns regarding the future of neurocognitive scientific activity on morphological processing (see also the General Commentary by Crepaldi and Amenta; doi: 10.3389/fpsyg.2013.00056).

As the (proud) Editors of this Research Topic, we honestly believe that the initial aims have been fulfilled. The excellence of the Original Research articles is doubtless, and they nicely cover different experimental approaches (i.e., behavioral or eye-tracking techniques, ERPs, TMS) to current questions regarding monolingual and bilingual lexical access. Similarly, the worth of the Review articles is undeniable. These Review articles represent a compelling updated overview of critical topics for the community investigating lexical access, and they will certainly serve for inspiration for other researchers in the field. Now it is time for the audience to assess the value of all these articles, and we sincerely hope that the reception will be at least as good as it has been during these last months, in which the amount of views and downloads of the articles has been heartening.
